# Disturbance of Key Cellular Subproteomes upon Propofol Treatment Is Associated with Increased Permeability of the Blood-Brain Barrier

**DOI:** 10.3390/proteomes10030028

**Published:** 2022-08-15

**Authors:** Timo Längrich, Kaya Bork, Rüdiger Horstkorte, Veronika Weber, Britt Hofmann, Matt Fuszard, Heidi Olzscha

**Affiliations:** 1 Institut für Physiologische Chemie, Martin-Luther-Universität Halle-Wittenberg, Hollystr. 1, 06114 Halle (Saale), Germany; 2 Klinik und Poliklinik für Herzchirurgie, Universitätsklinikum Halle (Saale), Ernst-Grube-Str. 20, 06120 Halle (Saale), Germany; 3 Core Facility—Proteomic Mass Spectrometry, Proteinzentrum Charles Tanford, Kurt-Mothes-Straße 3a, 06120 Halle (Saale), Germany; 4 Medical School Hamburg MSH, University of Applied Sciences and Medical University, Institute of Molecular Medicine, Am Sandtorkai 76, 20457 Hamburg, Germany

**Keywords:** anesthetics, blood-brain barrier, DNA damage response, drug effect, human brain microvascular endothelial cells, metabolic stress, propofol, proteome, quantitative proteomics, reactive oxygen species (ROS)

## Abstract

Background: Propofol is a short-acting anesthetic, which is often used for induction and maintenance of general anesthesia, sedation for mechanically ventilated adults and procedural sedation. Several side effects of propofol are known and a substantial number of patients suffer from post-operative delirium after propofol application. In this study, we analyzed the effect of propofol on the function and protein expression profile on a proteome-wide scale. Methods: We cultured human brain microvascular endothelial cells in absence and presence of propofol and analyzed the permeability of the blood-brain barrier (BBB) by fluorescein passage and protein abundance on a proteome-wide scale by mass spectrometry. Results: Propofol interfered with the function of the blood-brain barrier. This was not due to decreased adhesion of propofol-treated human brain microvascular endothelial cells. The proteomic analysis revealed that some key pathways in these cells were disturbed, such as oxygen metabolism, DNA damage recognition and response to stress. Conclusions: Propofol has strong effects on protein expression which could explain several side effects of propofol.

## 1. Introduction

The blood-brain barrier (BBB) is a semipermeable barrier that separates the peripheral blood from the brain parenchyma. It ensures that both endogenous substances and cells as well as exogenous substances and cells such as drugs or pathogens are prevented from entering the brain [1]. There are mainly three cell types that are the building blocks of the human BBB: the brain microvascular endothelial cells (BMECs), pericytes and astrocytes [2]. The BMECs are of particular importance for building a tight BBB, as they are pivotal in providing tight junctions and adherens junctions [3,4]. It has been reported that the rate of transcytosis in BMECs is comparably low, which leads to a tight and controlled BBB [5]. These polarized cells contribute to a tight paracellular and transcellular barrier which restricts the entry of the beforementioned components [6]. However, small lipophilic molecules can pass the BBB by the process of diffusion, whereas many larger hydrophilic molecules cannot pass this barrier and need to be transported via selectively expressed transport systems [7]. Transport systems ensure that molecules can be transported into the brain and waste products can be removed, especially, the so-called nutrient transporters help in transporting nutrients into the central nervous system (CNS) [8]. Since the brain is one of the organs with the highest energy-consumption, it is conceivable that nutrients and oxygen should not be the limiting factor. This is also reflected in the BMECs, as they express more mitochondria than comparable endothelial cells [9]. On the one hand, this can help the cells to fulfill their tasks in transporting and producing a sufficient concentration of molecules to maintain the barrier. On the other hand, the cells need a functioning system to remove excessive metabolites and even toxic products including reactive oxygen species (ROS), and a dysfunction of these systems could contribute to an increased permeability of the BBB [10].

Many pathological conditions are known to affect the integrity of the BBB, and some of them have been found to be associated with a disturbed oxygen metabolism. For instance, it has been shown that ROS and a disturbed oxygen metabolism affecting the BBB can deteriorate some neurodegenerative disorders such as Alzheimer’s disease (AD) and amyotrophic lateral sclerosis (ALS) [11,12]. However, it is still unclear, whether these phenomena are consequent reactions or causal in the etiology of these diseases. It is also still unclear, whether the effects of a disturbed oxygen metabolism are reversible and whether they could be treated.

Other than neurodegenerative disorders, there are other diseases known to be affected by decreased integrity of the BBB, for instance septic encephalopathy. It has been demonstrated that this condition can be associated with elevated protein levels in the cerebrospinal fluid and an increased uptake of different forms of iron oxide [13]. These phenomena are accompanied with increased permeability of the BBB and altered signal transduction in BMECs [14]. Cerebral ischemia is another pathologic condition which can lead to an increase in the permeability of the BBB [15,16], underpinning the hypothesis that altered oxygen levels and ROS can affect the integrity of the BBB. In the same vein, it has been reported that cerebral edema, which can occur after an ischemic stroke, can also have a significant impact on the tightness of the BBB, as the swelling of the cerebral tissue by the edema can culminate in an ischemic injury [17]. Other diseases which are also associated with an increased permeability of the BBB, are for instance meningitis and sepsis [18]. There are also indications that sepsis can be accompanied with post-operative delirium (POD), which can also contribute to the disruption of the BBB [19]. In the case of septic encephalopathy, patients display increased protein levels in the cerebrospinal fluid [20] and increased uptake of marked colloidal iron oxide has been reported in an animal model [21].

There exist several studies indicating that anesthesia can affect the BBB. One particular example we previously investigated was the non-volatile anesthetic propofol [22]. Propofol is a phenolic derivative and is considered one of the more favorable intravenous anesthetics due to its rapid onset of effect, short plasma half-life and its low accumulation in tissues [23]. Propofol is used for clinical anesthesia induction (4–6 μg/mL), maintenance of anesthesia and sedation (1–3 μg/mL) [24,25,26]. For instance, a concentration of 2.5 to 4 µg/mL is used during maintenance of anesthesia of normothermic cardiopulmonary bypass [27]. Other than the desired sedative effects, it causes hemodynamic [28], respiratory [29], neurological [30], endocrine [31] and immunogenic [32] effects. Propofol is a lipophilic compound, and can therefore rapidly cross the BBB. This also implies that propofol has to be dissolved in a lipophilic vehicle. Usually, it is formulated as an emulsion containing propofol, soybean oil (100 mg/mL), glycerol (22.5 mg/mL), egg lecithin (12 mg/mL) and disodium edetate (0.005%) with sodium hydroxide to adjust the pH to 7–8.5. This also implies that some, but not all of the side effects of propofol, can be explained by the accessory components in the emulsion, such as hyperlipidemia and also indicates the need for antimicrobial agents [33]. It has been previously shown that propofol can substantially reduce ischemia and reperfusion in particular by a decrease of reactive oxygen species (ROS), reduction of free radicals, helping in protection of the cell membrane and mitochondrial function from lipid peroxidation and a prevention of apoptosis [34,35,36]. It has been hypothesized that these mechanisms could contribute to an altered permeability of the BBB. However, there exist also reports showing that anesthetics including propofol did not alter the permeability of the BBB or even reduced an increased permeability during a 2% hypoxia [37]. In addition, there are indications that propofol may act in protecting DNA damage, including induced double strand breaks. Especially the ROS-induced DNA damage has been shown to be reduced by propofol treatment [38].

Considering our previous results and the aforementioned literature, we aimed with this current study to elucidate the effects of propofol on the BBB and systematically analyze changes in the proteome. With a previously established cellular model system [39], we demonstrated that propofol, but not its lipophilic vehicle formulation, leads to an increased permeability of the BBB model, whereas the cellular adhesion remained unchanged compared to the untreated control. Quantitative proteomic analysis by LC-MS/MS of the protein levels upon treatment with propofol revealed profound changes in biological processes (GO terms; [40,41]) such as oxygen transport, hydrogen peroxide processes, response to stress and DNA-damage recognition. We confirmed these results by biochemical means and could give evidence that the levels of representative proteins of these processes were altered. We could therefore give an explanation on a proteomic level for the previous observed effects of propofol on altered oxygen metabolisms, including the role of iron, DNA damage response (DDR) and a potential reversal of DNA damage. In addition, it could also explain some of the beforementioned long-term adverse effects. Since we clearly distinguished with our experiments between propofol itself and the lipidous emulsion, our results can give guidance on how to reduce the adverse effects.

## 2. Materials and Methods

### 2.1. Cells and Cell Culture

Transfected human brain microvascular endothelial cells (THBMEC), provided by MF Stins (Los Angeles, CA, USA), were used as model for BBB endothelial cells. DMEM/F12 medium (Thermo Fisher Scientific, Waltham, MA, USA) supplemented with 100 mg/L penicillin and 100 mg/L streptomycin (Thermo Fisher Scientific), 2 mM L-glutamine (Thermo Fisher Scientific) and 10% heat-inactivated fetal calf serum (GE Healthcare, Little Chalfont, UK) was used for cultivation at 37 °C in a humified cell culture incubator. Cells were passaged every 2–3 days. Therefore, THBMECs were detached with 1% Trypsin/EDTA (Thermo Fisher Scientific) and pelleted at 210 g for 5 min. A more detailed description of the method is given in [39].

### 2.2. Measurement of Fluorescein Passage through the BBB

Testing the effect of propofol treatment on the permeability of the human BBB was performed by an endothelial cell culture model, which mimics a tight BBB model with human brain microvascular endothelial cells (THBMEC) [42]. THBMECs were grown on 12-well filters with 3.0-µm pore size (ThinCerts, Greiner Bio-One, Kremsmünster, Austria) for 14 days until they form a confluent layer. Firstly, filters were coated with a mixture of 10 µg/mL collagen IV and 10 µg/mL fibronectin (Sigma-Aldrich, Saint Louis, MO, USA) for 24 h. Incubation of the cells was performed using a cell culture incubator with 5% CO_2_ atmosphere at 37 °C. DMEM/F-12 medium was changed every 2 to 3 days in the upper and lower chamber. Propofol (Sigma-Aldrich) was dissolved in lipid mixture (SMOFlipid, FreseniusKabi, Bad Homburg, Germany). On day 14 of growth, propofol was added to serum free medium in the upper chamber to a final concentration of 3 µg/mL, an equal amount of the lipid mixture served as a control. After 24 h of treatment, the medium was removed, cells were washed two times with PBS and serum free medium without phenol red was given into both chambers. Fluorescein was added to the upper chamber, with a final concentration of 10 µg/mL. After an incubation time of 180 min, aliquots were taken from lower chamber and fluorescence intensity was measured (Clariostar, BMG Labtech GmbH, Ortenberg, Deutschland) at a wavelength of 535 nm (excitation: 488 nm). A calibration curve was produced by using a dilution series with known concentration of fluorescein (2 ng/mL to 1 mg/mL).

### 2.3. Real Time Cell Adhesion Assay

xCelligence RTCA DP^®^ in combination with E-Plates^®^ (OMNI Life Science, Bremen, Germany) was used for time-dependent measurement. On a first step E-Plates^®^ were coated with 10 µg/mL collagen IV and 10 µg/mL fibronectin for 72 h. Plates were washed twice with PBS and then blocked with 0.5% bovine serum albumin (Carl Roth, Karlsruhe, Germany) in PBS for 30 min at 37 °C. Cells were detached with 1% Trypsin/EDTA and counted while wells were equilibrated for 15 min with 50 µL medium at 37 °C. After incubation time, the plates were set into RTCA DP^®^ and blank was measured. Afterwards 2500 cells were seeded in every well and propofol was given directly into medium, untreated cells and cells treated with lipid served as a control. Cell index was measured automatically every 15 min.

### 2.4. Preparation of Cell Extracts

After incubation, treated and control cells were removed from the surface by scraping and washed twice in ice cold PBS. Lysis buffer was prepared by supplementing solubilization buffer (50 mM Tris pH = 7.4, 150 mM NaCl, 1% Triton X-100, 1% SDS (sodium dodecyl sulfate), 1 mM EDTA (ethylenediaminetetraacetic acid)) with 0.2% PIC (protease inhibitor cocktail), 0.1% PMSF (phenylmethylsulfonylfluoride), 0.05% Mg132, 1% Sodium orthovanadate, each 1% phosphatase inhibitor cocktail II and III, 5 µM SAHA (suberoylanilide hydroxamic acid) and 400 µM NEM (*N*-ethylmaleimide). Protein concentration was measured using BCA (bicinchoninic acid assay, Thermo Scientific Fisher) following the manufacturer’s instructions.

### 2.5. Mass Spectrometry

#### 2.5.1. Sample Preparation

First, 10 µg of protein from each sample were aliquoted and the remaining sample stored at −20 °C. Samples were then prepared for mass spectrometry analysis with the USP3 protocol as described [43]. In brief, 1 µL of pre-prepared Sera-Mag speed beads were added to each sample and vortexed. Acetonitrile (ACN) was added to 70% and the mixture incubated for 20 min at RT in a thermo mixer at 400 rpm to allow proteins to adsorb to the beads. Samples were placed on a magnetic rack for 2 min and the supernatant was discarded. Samples were subsequently washed three times on-bead with 70% ethanol and finally with pure ACN. After lyophilizing the beads to remove ACN, samples were re-suspended in 50 mM Ammonium Bicarbonate and reduced with 10 nM DTT at 80 °C for 15 min. After cooling samples were alkylated with 20 mM CAA for half an hour at room temperature in the dark. LysC/trypsin was added at a ratio of 1:50 enzyme:substrate and incubated overnight at 37 °C. Samples were then placed on a magnetic rack for 2 min and the supernatant with peptides were removed for C18 spin column clean up (Pierce^TM^ C18 Spin Tips, Thermo Scientific #84850) as recommended by the manufacturer.

#### 2.5.2. LC-MSMS

Approximately 200 ng of peptides for each sample and replicate were initially trapped (PepMap100 5 µm, 3 × 5 mm Thermo Scientific #160454) and separated on a Waters M-Class C18 25 cm analytical column (Acquity UPLC^®^ M-Class HSS T3 1.8 µm 75 µm × 250 mm, Waters #18600) over 180 min with an increasing gradient of ACN (3–22%) at 240 nL/min before being injected in to a Thermo Scientific Orbitrap Exploris 480 mass spectrometer. Peptides were ionized in positive mode with 1800 V and a transfer capillary temperature of 300 °C. Samples were subjected to further separation with FAIMS pro with 3 compensation voltages (CVs: −40, −55, −65), resulting in 3 separate MS experiments within one data file. Each experiment had the following settings: MS resolution of 120,000 at 00 m/z, a scan range of 350–1400 m/z, MS AGC target of 300% for max IT of 50 ms; MSMS of all the most intense peaks for a total cycle time of 1 s with the following settings: Isolation window of 2 m/z normalized collision energy of 30%, resolution of 15 000 with an AGC target of 100% and a max 30 ms IT. Every fragmented precursor within +/−10 ppm was immediately excluded from reanalysis for 45 s.

#### 2.5.3. Data Analysis

Raw data files were analyzed within Proteome Discoverer 2.4.0.305 following a LFQ quantification workflow. Searches were performed against the Human database (accessed 7 July 2020—Uniprot proteome UP000005640) with decoys using Sequest HT with a precursor tolerance of 10 ppm and a fragment mass tolerance of 0.02 Da, trypsin as a cleavage agent with 2 missed cleavages considered. Carbamidomethylation of cysteines was set as a static modification, and the following dynamic modifications were investigated: oxidation (M), acetylation (K), phosphorylation (S, T, Y), ubiquitination (GG on K), as well as N-terminal protein modifications of acetylation, met-loss and acetylation + met-loss. IDs were filtered with Percolator at a strict FDR of 0.01. Precursor ion quantification was performed with unique and razor peptides with Top N of 3. Precursor abundance was based upon intensity and normalized over total peptide amount. Quantification was based on ratios of lipid:control and propofol:control where pairwise ratios were calculated and *t*-tests were used for hypothesis testing [43].

The mass spectrometry proteomics data have been deposited to the ProteomeXchange Consortium via the PRIDE [44] partner repository with the dataset identifier PXD033856 and 10.6019/PXD033856.

### 2.6. Immunoblotting

Cell lysates were supplemented with SDS-sample buffer (100 mL buffer containing 12.5% SDS, 0.3 M Tris, 50 mL glycerin, bromophenol blue at pH 6.8 and 1:10 DTT to buffer) and heated at 95 °C for 10 min. Then, 40 µg of each sample were applied on a 12% acrylamide gel and separated by SDS-PAGE. Afterwards proteins were transferred to a nitrocellulose membrane for 1 h 15 min in blotting buffer with a constant amperage of 300 mA. To avoid overheating, blot chamber (VWR, Radnor, PA, USA) was cooled down during the blotting process. To prove successful protein transfer staining with ponceau red solution containing 0.1% ponceau S (Carl Roth, Karlsruhe, Germany), 3% trichloroacetic acid and 3% sulfosalicylic acid was applied. Before incubating the membrane with the primary antibodies, it was blocked with 5% milk in 1 × TBS supplemented with 0.05% Tween-20 (TBS-T) at room temperature for 1 h. Incubation with primary antibodies was performed overnight at 4 °C. Histone H2AX was detected by rabbit monoclonal Anti-Histone H2AX antibody (ab229914, Abcam, Cambridge CB2 0AX, UK) at a 1:1000 dilution. FTH1 was detected using FTH1 Rabbit monoclonal antibody (CST 4393, Cell Signaling Technologies, Danvers, MA, USA) at a dilution of 1:1000. The next day the membrane was washed three times with TBS-T for 15 min and then incubated with HRP-conjugated goat-anti-rabbit-antibody (ab6721, Abcam), diluted 1:20,000, at room temperature for 1 h. Antibody binding to specific proteins were detected by using Luminata Forte Western HRP-Substrate (Merck Millipore, Billerica, MA, USA) and signals were visualized using the ChemiDoc MP Imaging System (BioRad, Hercules, CA, USA). Analysis was performed by using the associated ImageLab software (BioRad). For normalization of band intensity of the Western blot Ponceau S staining served as the loading control. Comparison and *p*-value determination of two samples in the quantitated Western blots was performed by using a paired student’s *t*-test.

## 3. Results

In order to quantify the effects of propofol on the permeability of the BBB for compounds from the blood, we used our recently established BBB-model, which takes advantage of human brain endothelial cells (THBMEC) [39]. These cells were grown on filters until confluence and prevent diffusion of molecules from the upper to the lower side of the filter.

### 3.1. Propofol Increases the Permeability of the BBB

**Figure 1:**
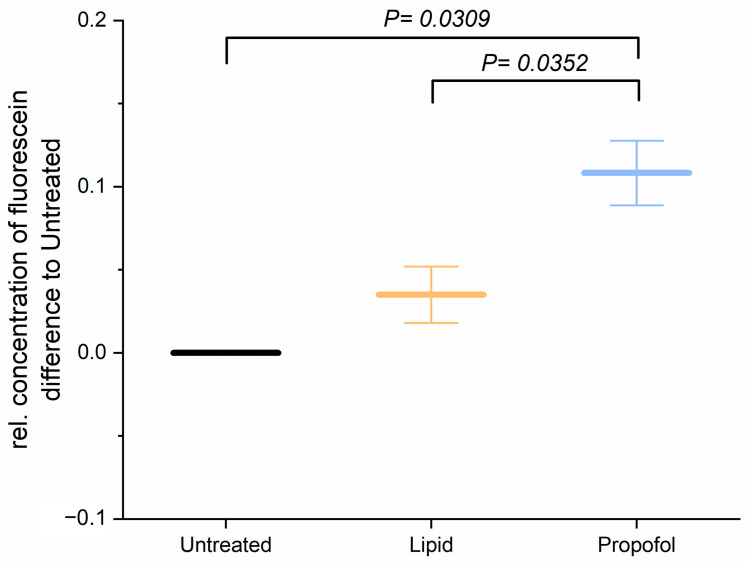
Cells were treated with 3 µg/mL propofol, dissolved in lipid solution, for 24 h. Lipid treatment and untreated cells served as control. Fluorescein was applied to the upper chamber and the concentration in lower chamber was quantified after 1 h. The bar chart represents mean of relative concentration of fluorescein ± SD (*n* = 3).

Before studying the permeability of the BBB after application of propofol, we analyzed the cell viability of THBMEC cells in the presence of propofol with 3 µg/mL, which is a commonly used concentration in surgery, and found that the cell viability was not affected by propofol (data not shown and in [22]). The effect of propofol on the permeability of THBMECs was further analyzed. Treatment with 3 µg/mL propofol resulted in an increase of the permeability of the endothelial cells (Figure 1), whereas the lipid solution, in which propofol is dissolved, had no effect.

**Figure 2:**
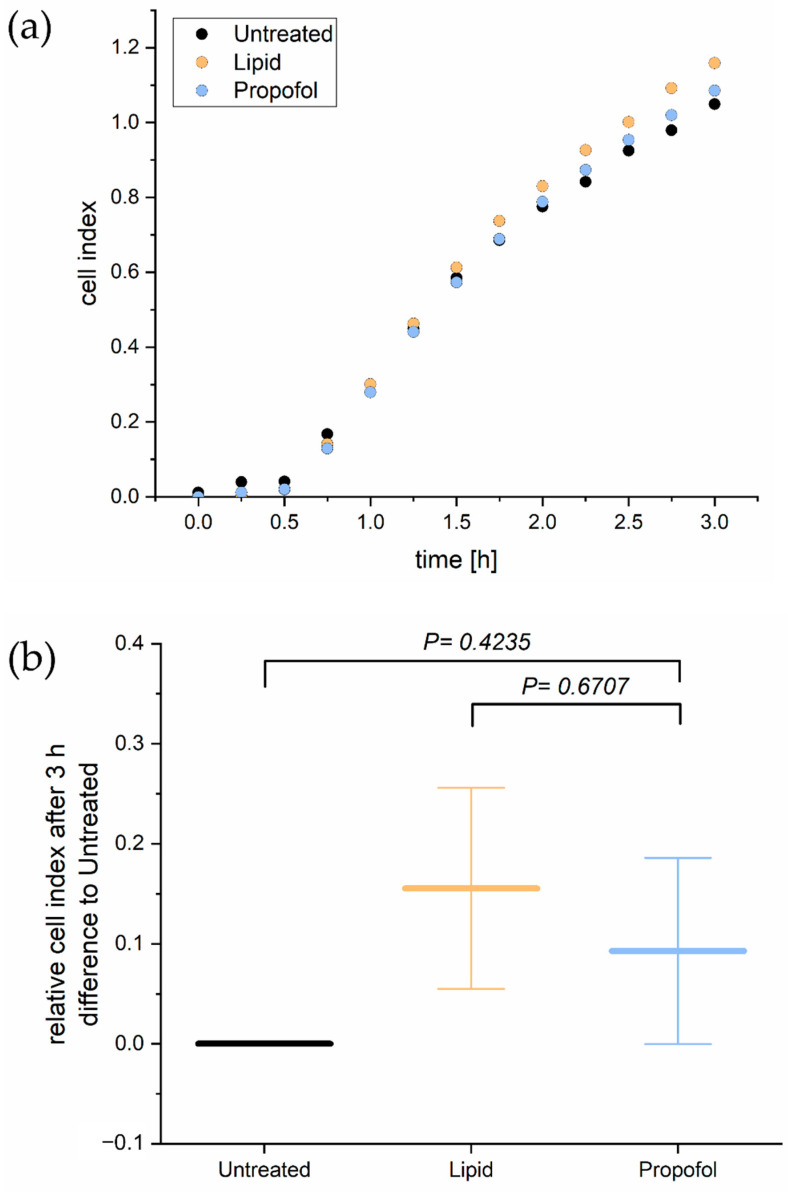
Real time cell adhesion assay. Cells were treated with 3 µg/mL propofol. Cell adhesion was measured every 15 min for 3 h (**a**). The bar chart represents differences of the mean cell index compared to propofol treated cells and cells treated with the only the lipid vehicle ± SD after 3 h (**b**), (*n* = 3).

Since tightness and permeability of the BBB rely mainly on cell adhesion, we analyzed next the adhesion of THBMECs on the surface. In order to determine the cellular adhesion, we performed a real-time cell adhesion assay. The time course of one representative experiment is shown in Figure 2A. There was no significant difference in adhesion of THBMECs in the absence or presence of propofol over 3 h of adhesion. Figure 2B shows the relative mean adhesion of three independent experiments after 3 h.

### 3.2. Propofol Interferes with Protein Expression of the BBB

**Figure 3:**
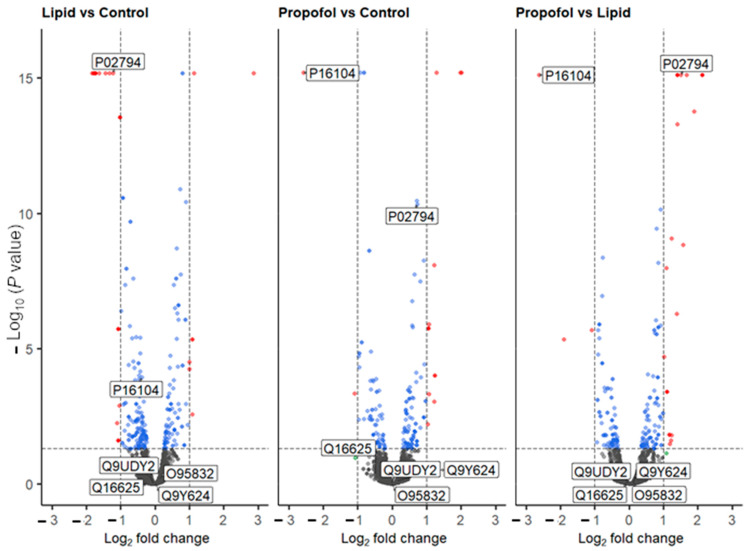
Volcano plot of all quantified proteins from the three different comparative experiments. Proteins that are up- or down-regulated are shown in red. Labels are Uniprot codes, whereby P16104 has the gene name H2AFX (protein name H2AX), and P02794 is FTH1. Dotted lines represent significant differences from control and is given by *p* < 0.05, and fold change (log2) > 2. Blue dots represent data points that have passed the *p* value threshold and the red dots are data points that have passed both the *p* value and fold change thresholds listed above.

We then asked the question: why propofol interferes with the permeability of the BBB without interfering neither cell viability nor cell adhesion? To answer this question, we analyzed the protein abundance of THBMECs cultured in the absence or presence of propofol by quantitative mass spectrometry. We were able to identify 6903 protein groups across 3 conditions (*n* (replicates per condition) = 3). Of these, 5440 proteins were quantifiable (FDR < 0.01; #unique peptides ≥ 2, *n* = 9). The volcano plots of the differentially abundant proteins are given in Figure 3. Lipid vs. control saw 23 proteins that were differentially abundant (17 down, 6 up), propofol vs. control had 22 proteins (12 down, 10 up) and propofol vs. lipid had 24 (5 down, 19 up) (Supplementary Tables S1–S4, Supplementary Figure S1).

Based on the MS data, we analyzed the protein levels of two specific proteins in more detail, namely the histone H2AX (P16104, gene name *H2AFX*, Figure 3), which was found to be down-regulated in the MS analysis after propofol vs. control (FC = −2.58, *p* < 0.001) and propofol vs. lipid (FC = −2.62, *p* < 0.001) (but not lipid vs. control, but was mildly depressed (FC = −0.39, *p* < 0.001)) and the heavy chain subunit FTH1 (P02794, Figure 3) of ferritin, which was significantly up-regulated upon propofol vs. lipid (FC = 1.5, *p* < 0.001), but not significantly different in propofol vs. control (FC = 0.72, *p* < 0.001) and down-regulated in lipid vs. control (FC = −1.23, *p* < 0.001).

**Figure 4:**
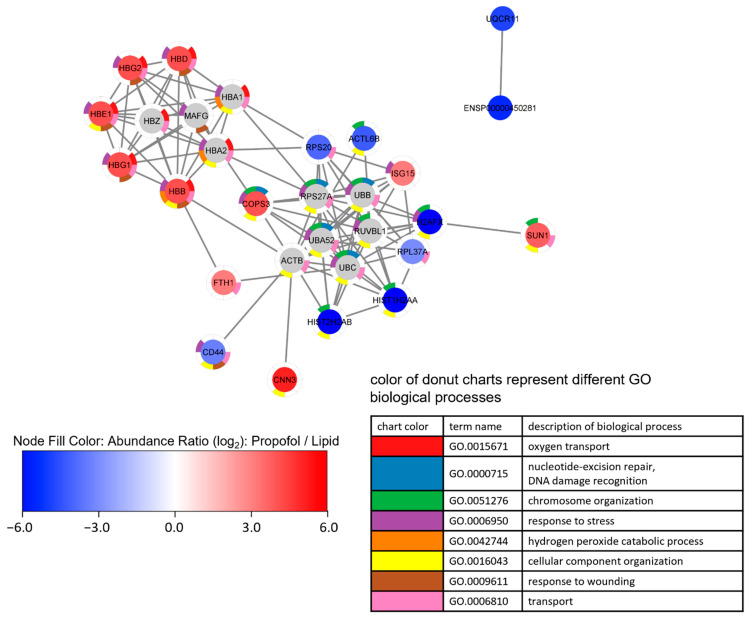
Network analysis of a proteome network after propofol treatment. Cells were treated with 3 µg/mL propofol for 12 h, lipid treatment served as a control. Total protein was isolated and analyzed by mass spectrometry (*n* = 3). Proteins are filtered according following parameter: (1) 1.5-fold increase or decrease in abundance ratio (log_2_)—propofol vs. lipid; (2) coverage ≥ 5%; (3) protein found in every sample.

We also performed a cluster analysis by String implemented in Cytoscape in order to find interactions between differently expressed proteins. Up- and down-regulated proteins form different clusters are depicted in Figure 4. Looking at the enriched terms of GO biological processes, oxygen transport and response to stress are found in up-regulated proteins, while down-regulated proteins are associated with chromosome organization.

Proteins were clustered by String via Cytoscape using up to 10 interactors, followed by an enrichment analysis. Node fill color represents abundance ratio (log_2_)—propofol vs. lipid, interactors added by String are colored grey. The donut chart represents the eight most significant terms of GO biological processes found in the enrichment analysis, which is reflected in the legend. Proteins without annotated interactions are not shown.

**Figure 5:**
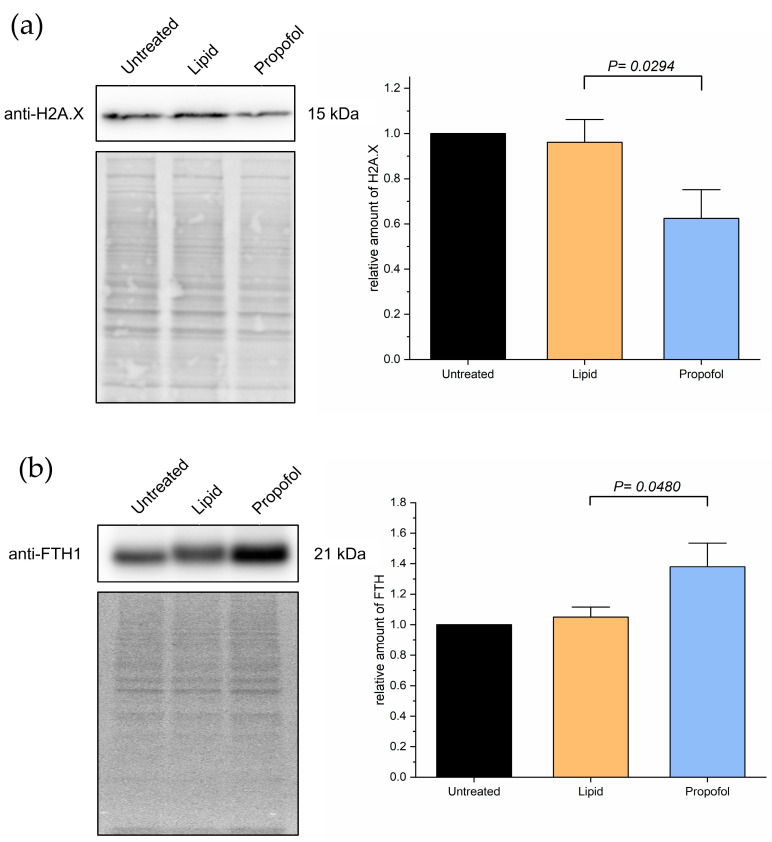
Cells were treated with 3 µg/mL propofol for 48 h, untreated cells and lipid treatment served as control. Afterwards, total protein was isolated and separated using SDS-PAGE. Expression of proteins was detected via immuno-blotting using anti-H2AX-antibody (**a**) and anti-FTH-antibody (**b**) (*n* = 4). Ponceau S served as a loading control. Bar chart represents mean of amount of target protein ± SD relative to untreated cells.

Given these observations in the MS experiments, and given the importance of the aforementioned proteins to the processes in Figure 4, we validated the changing protein levels of these two proteins by Western blot analysis as shown in Figure 5. We found a substantial reduction of H2AX protein expression compared to control (Figure 5A) and a slight increase in FTH1 protein expression (Figure 5B), which correlates with the data obtained by MS. The comparative differences of both proteins in expression are clearly shown in propofol vs. lipid (Figure 4) and the Western blots.

## 4. Discussion

In this study, we could demonstrate that propofol affects the blood-brain-barrier on human endothelial cells. Importantly, it directly reduces its function by increasing the permeability for metabolites and artificial compound from the apical side, where nutrients and oxygen would be provided. In order to elucidate additional effects and give an explanation for the observed increased permeability in the BBB, we performed a quantitative MS analysis of untreated cells, cells treated with propofol in its vehicle and cells treated with the vehicle only. The experiments provided evidence that propofol treatment leads to changes in the proteome of human endothelial brain cells, particularly of pathways involved in metabolic stress, ROS metabolism and DNA damage response (DDR) and recognition.

H2AX is a histone protein from the H2A family, which is activated by phosphorylation. It modulates nucleosome-formation, chromatin-remodeling and is involved in DNA repair. It is a marker for double-strand breaks in dsDNA and it interacts with several other proteins which are involved in DNA repair, such as BRCA1 or MDC1. It has been demonstrated that H2AX plays a role in the transcription of genes regulated by FoxO3a, which is associated with aging/longevity and genomic instability [45]. Therefore, it is possible that down-regulation of H2AX by treatment with propofol has longer lasting effects, which we have been observed in the decreased function of the blood-brain-barrier.

Ferritin has been identified as an iron transporter of the blood-brain-barrier [46]. Upregulation of FTH1 via propofol treatment could therefore increase the iron concentration in the brain, and it has been demonstrated that aging goes along with increased iron concentrations in the brain. In addition, the accumulation of iron in neurons induces damage by apoptosis [47]. The up-regulation of FTH1 observed in our experiments could lead to toxic iron concentrations in the brain, what could explain at least some of the long-lasting side effects of propofol.

Based on the MS results, we could also conclude that the affection of the BBB is most likely an indirect effect, because no cell adhesion molecules have been found among the significantly up-/down-regulated proteins in MS analysis. This means that presence of cell adhesion molecules, which are responsible for the function of the BBB, is not changed among the proteins which were detected. Since we analyzed the function after relatively short time periods (hours), we could only see changes in the given time window and this may explain that we could not observe a direct effect on proteins which are responsible in building the BBB. The observed changes in protein levels have most likely long-lasting effects and could have dramatic side effects for patients. Further investigation is necessary to clarify if maybe the intracellular localization of cell adhesion molecules is affected instead of changes in protein levels. For instance, we cannot exclude changes in posttranslational modifications (PTMs), and it has been reported that a change in PTMs can affect the BBB substantially [48]. That does not only include commonly seen PTMs such as phosphorylation [49] or ubiquitination [50], but also for instance palmitoylation [51]. Since we could see a decrease in H2AX levels upon propofol treatment, it would be interesting to analyze the phosphorylation status of this protein. Since we did not enrich for particular PTMs, such as phosphorylation, we would like to investigate these questions in a potential follow-up study. In addition, ROS can also lead to a direct damage of molecules responsible for forming tight barriers, which underpins the current findings [52]. Furthermore, we could show in a previous study that glycation as it is observed during a severe diabetes mellitus leads in combination with the treatment of propofol to an additive effect regarding an increased permeability of the BBB [22]. In line with this observation, it is likely that other proteins which were detected to be changed after treatment with the vehicle or propofol plus the vehicle are affected by PTMs. For instance, we noticed a slight change of FTH1 towards a higher molecular weight upon treatment with the compounds. This is consistent with previous observations reporting phosphorylation on at least two serine residues of FTH1 [53,54], and could indicate increased phosphorylation in addition to an upregulation of the protein.

We expected to see the level of proteins changed, which are directly involved with building the BBB, for instance occludins, claudins and ZO-proteins. Interestingly, proteins directly involved in building the BBB were shown to be enriched in our pathway analysis. There are several possibilities as to why we do not see a direct effect. Firstly, the effects may take more than a day to show substantial and detectable changes in their levels and will likely not reflect all changes of a complex proteome. Secondly, the effects on the BBB could be dependent on changes of the PTMs. There is evidence that PTMs can modulate the barrier functions in epithelial cell in general and epithelial cells building the BBB in particular. It has been shown that not only claudins are modified for instance by phosphorylation, palmitoylation ubiquitination and SUMOylation, but also occludins, tricellulins and angulins [55].

In addition, the performed experiments give strong indications that not only propofol can affect the BBB, but also the lipidous vehicle may be able to affect the permeability of the BBB. Propofol is an anesthetic with favorable properties, however, the formulation with lipidous vehicles such as soybean oils has some disadvantages, including emulsion instability, injection pain, a need for antimicrobial agents to prevent sepsis and potential hyperlipidemia-related side effects [33]. Here, we could clearly distinguish in the proteomic profile between the effects of propofol and the respective vehicle. It also underpins the need to develop different, more favorable vehicles with less side effects.

It is obvious that metabolic stress, ROS metabolism and DDR are interconnected, and it may give an explanation for certain observed long-term actions of propofol such as impairment of cognitive function, associated with neuroapoptosis [56,57]. It may well be that an increase of metabolic stress and ROS contribute to apoptosis not only in endothelial cells of the BBB, but also in neurons. In addition, it has been shown that propofol also inhibits long-term potentiation in a rat model, suggesting it has a strong effect on the nervous system potentially via the BBB. Given the potential effect of ROS-species leading to a collapsed BBB, treatment with compounds reducing oxidative stress during and after treatment with propofol may be beneficial. This hypothesis supports our previous observation that a less tighter BBB upon glycation may be partially reduced upon treatment with ascorbic acid, colloquially known as vitamin C [22].

## 5. Conclusions

Propofol treatment leads to an increased permeability of the BBB. This is accompanied by changes of subproteomic networks associated with metabolic stress and proteins involved in ROS metabolisms as well as DDR. These results may explain some of the long-term effects of propofol on the central nervous system and underpins the benefit of compounds acting as scavengers such as ascorbic acid.

## Data Availability

The mass spectrometry proteomics data have been deposited to the ProteomeXchange Consortium via the PRIDE [44] partner repository with the dataset identifier PXD033856 and 10.6019/PXD033856.

## Supplementary Materials

The following are available online at https://www.mdpi.com/article/10.3390/proteomes10030028/s1, Figure S1: PCA plot, Table S1: Differentially_quantified_proteins, Table S2: Peptides, Table S3: Protein, Table S4: PSM.
