# Supplementary material for: Disturbance of Key Cellular Subproteomes upon Propofol Treatment Is Associated with Increased Permeability of the Blood-Brain Barrier
Source: Proteomes. 2022 Aug 15;10(3):28. doi: 10.3390/proteomes10030028 (PMC9397097; doi:10.3390/proteomes10030028)
Supplement: Supplementary file 1 [file proteomes-10-00028-s001.zip › Supplementary Figure S1 PCA plot.pdf]

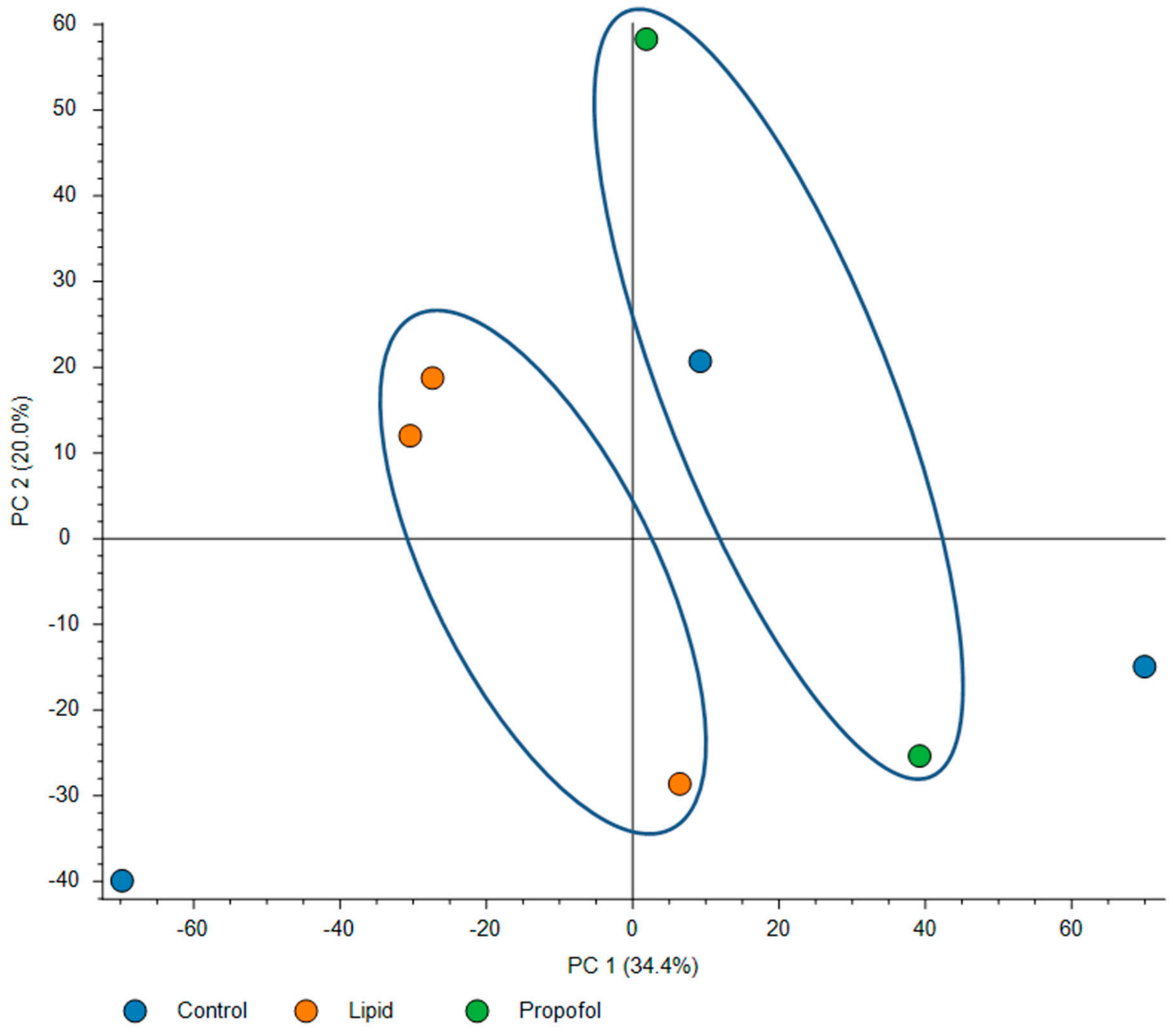

Figure S1. PCA plot.

Supplementary Figure Legend S1

Principal component analysis (PCA) of biological replicates of control, lipid and propofol treatment.
